# (2,2′-Bi­pyridine-κ^2^ *N*,*N*′)bis­(nitrato-κ^2^ *O*,*O*′)copper(II)

**DOI:** 10.1107/S1600536813028201

**Published:** 2013-10-19

**Authors:** Feng-Yi Liu, Ming-Hui Zhang, Jun-Feng Kou

**Affiliations:** a College of Chemistry and Chemical Engineering, Yunnan Normal University, Kunming 650500, People’s Republic of China

## Abstract

In the title complex, [Cu(NO_3_)_2_(C_10_H_8_N_2_)], the Cu^II^ cation is chelated by two nitrate anions and by one 2,2′-bi­pyridine ligand in a distorted N_2_O_4_ octa­hedral geometry. The dihedral angle between the pyridine rings is 1.92 (11)°. In the crystal, π–π stacking between parallel pyridine rings of adjacent complex mol­ecules is observed, the centroid–centroid distance being 3.6788 (19) Å. Weak C—H⋯O hydrogen bonds further link the mol­ecules into a three-dimensional supra­molecular architecture.

## Related literature

For applications of copper(II) complexes in magnetochemistry, see: Garribba *et al.* (2000 ▶); Mukherjee (2000 ▶).
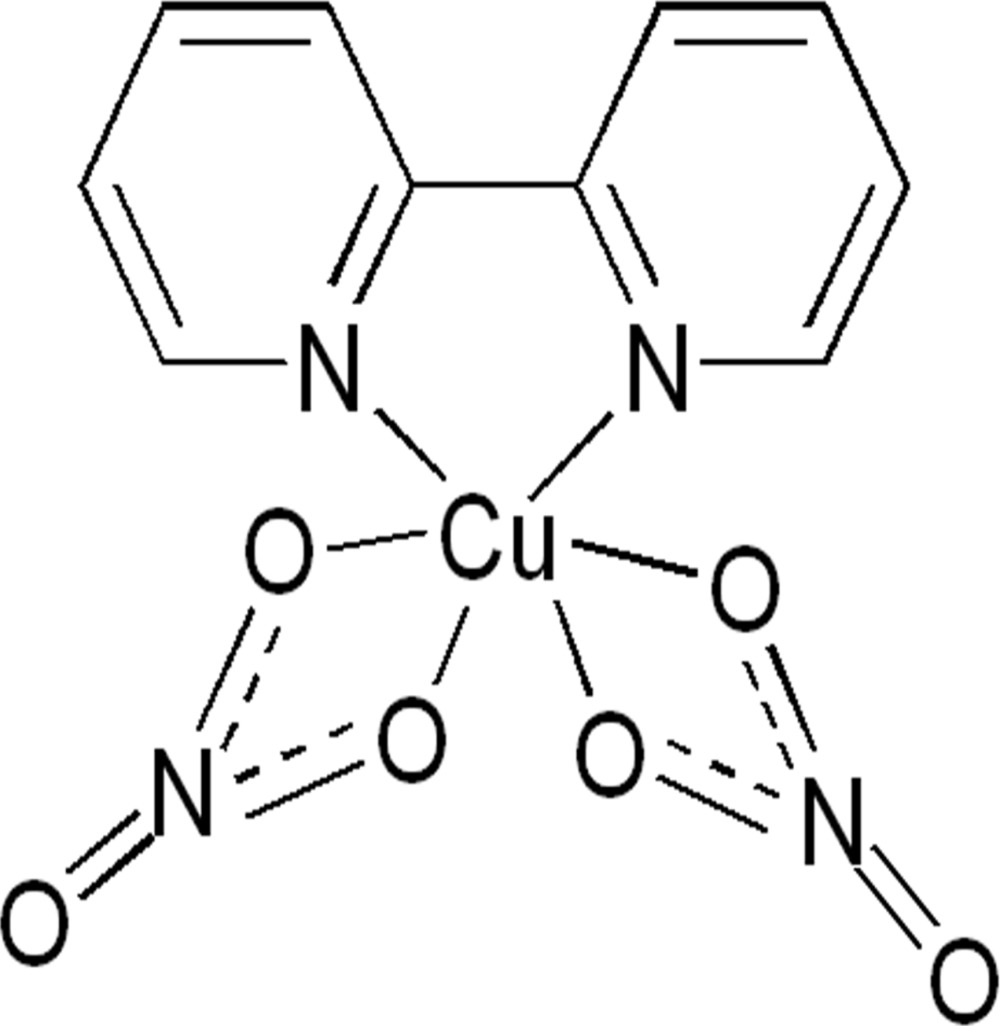


## Experimental

### 

#### Crystal data

[Cu(NO_3_)_2_(C_10_H_8_N_2_)]
*M* *_r_* = 343.74
Monoclinic, 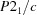

*a* = 8.4282 (17) Å
*b* = 11.132 (2) Å
*c* = 16.140 (5) Å
β = 121.39 (2)°
*V* = 1292.7 (5) Å^3^
*Z* = 4
Mo *K*α radiation
μ = 1.73 mm^−1^
*T* = 293 K
0.30 × 0.28 × 0.25 mm

#### Data collection

Rigaku MM007-HF CCD (Saturn 724+) diffractometer
12333 measured reflections
2955 independent reflections
2237 reflections with *I* > 2σ(*I*)
*R*_int_ = 0.038

#### Refinement

*R*[*F* ^2^ > 2σ(*F* ^2^)] = 0.035
*wR*(*F* ^2^) = 0.098
*S* = 1.04
2955 reflections
190 parameters
7 restraints
H-atom parameters constrained
Δρ_max_ = 0.44 e Å^−3^
Δρ_min_ = −0.45 e Å^−3^

### 

Data collection: *CrystalStructure* (Rigaku/MSC, 2006 ▶); cell refinement: *CrystalStructure*; data reduction: *CrystalStructure*; program(s) used to solve structure: *SHELXTL* (Sheldrick, 2008 ▶); program(s) used to refine structure: *SHELXTL*; molecular graphics: *DIAMOND* (Brandenburg, 1999 ▶); software used to prepare material for publication: *SHELXTL*.

## Supplementary Material

Crystal structure: contains datablock(s) I, new_global_publ_block. DOI: 10.1107/S1600536813028201/xu5745sup1.cif

Structure factors: contains datablock(s) I. DOI: 10.1107/S1600536813028201/xu5745Isup2.hkl

Additional supplementary materials:  crystallographic information; 3D view; checkCIF report

## Figures and Tables

**Table 1:** Selected bond lengths (Å)

Cu1—N1	1.966 (2)
Cu1—N2	1.970 (2)
Cu1—O1	2.411 (2)
Cu1—O2	1.994 (2)
Cu1—O4	2.437 (2)
Cu1—O5	1.9987 (19)

**Table 2:** Hydrogen-bond geometry (Å, °)

*D*—H⋯*A*	*D*—H	H⋯*A*	*D*⋯*A*	*D*—H⋯*A*
C1—H1⋯O1^i^	0.93	2.56	3.390 (3)	149
C4—H4⋯O5^ii^	0.93	2.50	3.422 (3)	169

Symmetry codes: (i) 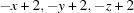; (ii) 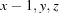.

**Fig. 1.**
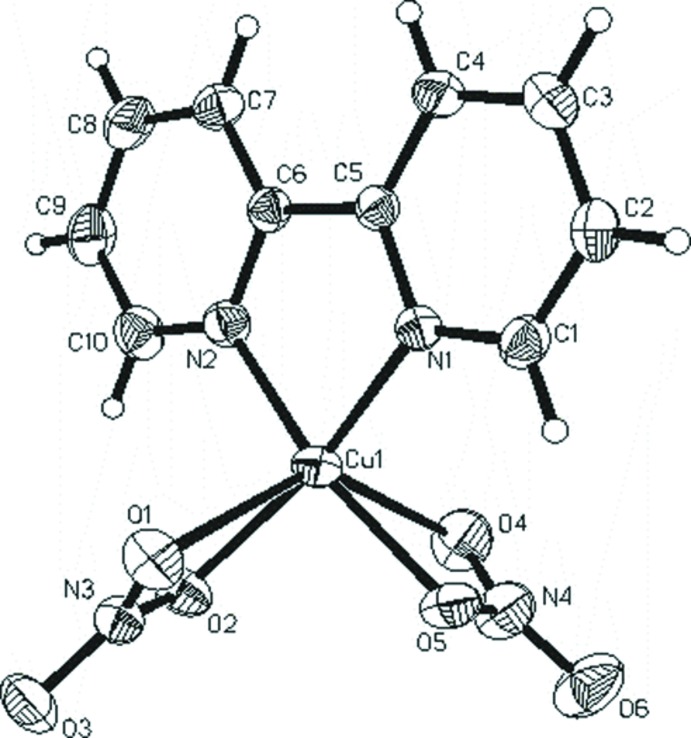
The molecular structure of the title compound, with atom labels and 30% probability displacement ellipsoids.

**Fig. 2.**
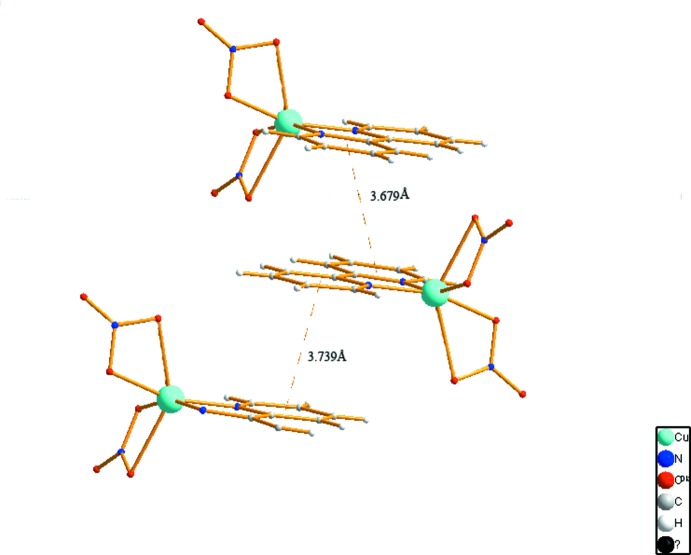
The pi···pi stacking between two asymmetric cations.

**Fig. 3.**
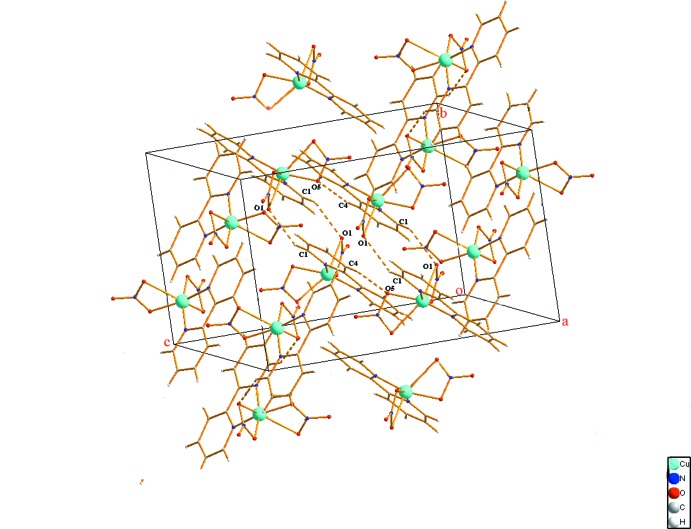
A view of the crystal packing. Hydrogen bonds are shown as brown dashed lines.

## supplementary crystallographic information

### 1. Comment

Copper(II) is one of the most important transition metals in magnetochemistry (Garribba *et al.* 2000; Mukherjee, 2000). Herein we report the synthesis and structure of the title copper(II) complex with 2,2'-bipyridine.

As shown in Fig.1, the Cu(II) atom is chelated by two N atoms of 2,2'-bipyridine and four O atoms of from two nitrate anions, forming an irregular octahedral coordination geometry. The Cu—N bond distances are 1.9661 (19) Å and 1.9691 (18) Å with basal angle of 82.48 (8). The apical positions are occupied by O atoms of the two different bis-chelating nitrate anions [Cu—O distances of 2.4100 (19) Å, 1.9948 (18) Å, 2.28 (3) Å and 1.9983 (16) Å) with an angle of 57.76 (7), 91.89 (8) and 55.9 (7). The dihedral angle between the planes of the two pyridine rings is 1.92 (11)°. Further, π–π stacking interactions with a centroids separation of 3.6788 (19) Å between pyridine rings and weak C1—H1···O1 and C4—H4···O5 hydrogen bonds link the molecules into the three dimensional supramolecular structure in Fig. 2 and Fig. 3.

### 2. Experimental

A solution of copper(II) nitrate hydrate (0.2 mmol, 48 mg) in methanol (2 ml) was mixed with 2 ml of an aqueous solution of *p*-aminobenzoic acid (0.1 mmol, 17 mg) in presence of 2,2-bipyridine (0.1 mmol, 16 mg). The resulting mixture was allowed to evaporate for one week to yield a blue crystal, suitable to X-ray work.

### 3. Refinement

H atoms were geometrically fixed and allowed to ride on the non-H atom with C—H = 0.93 Å, *U*_iso_(H) = 1.2*U*_eq_(C).

### Crystal data

**Table:**

[Cu(NO_3_)_2_(C_10_H_8_N_2_)]	*F*(000) = 692
*M**_r_* = 343.74	*D*_x_ = 1.766 Mg m^−^^3^
Monoclinic, *P*2_1_/*c*	Mo *K*α radiation, λ = 0.71073 Å
Hall symbol: -P 2ybc	Cell parameters from 2955 reflections
*a* = 8.4282 (17) Å	θ = 3.0–27.5°
*b* = 11.132 (2) Å	µ = 1.73 mm^−^^1^
*c* = 16.140 (5) Å	*T* = 293 K
β = 121.39 (2)°	Block, blue
*V* = 1292.7 (5) Å^3^	0.30 × 0.28 × 0.25 mm
*Z* = 4	

### Data collection

**Table:**

Rigaku MM007-HF CCD (Saturn 724+) diffractometer	2237 reflections with *I* > 2σ(*I*)
Radiation source: rotating anode	*R*_int_ = 0.038
Confocal monochromator	θ_max_ = 27.5°, θ_min_ = 3.0°
ω scans at fixed χ = 45°	*h* = −10→10
12333 measured reflections	*k* = −14→14
2955 independent reflections	*l* = −20→18

### Refinement

**Table:**

Refinement on *F*^2^	Primary atom site location: structure-invariant direct methods
Least-squares matrix: full	Secondary atom site location: difference Fourier map
*R*[*F*^2^ > 2σ(*F*^2^)] = 0.035	Hydrogen site location: inferred from neighbouring sites
*wR*(*F*^2^) = 0.098	H-atom parameters constrained
*S* = 1.04	*w* = 1/[σ^2^(*F*_o_^2^) + (0.050*P*)^2^ + 0.3548*P*] where *P* = (*F*_o_^2^ + 2*F*_c_^2^)/3
2955 reflections	(Δ/σ)_max_ = 0.002
190 parameters	Δρ_max_ = 0.44 e Å^−^^3^
7 restraints	Δρ_min_ = −0.45 e Å^−^^3^

### Special details

**Table:**

Geometry. All e.s.d.'s (except the e.s.d. in the dihedral angle between two l.s. planes) are estimated using the full covariance matrix. The cell e.s.d.'s are taken into account individually in the estimation of e.s.d.'s in distances, angles and torsion angles; correlations between e.s.d.'s in cell parameters are only used when they are defined by crystal symmetry. An approximate (isotropic) treatment of cell e.s.d.'s is used for estimating e.s.d.'s involving l.s. planes.
Refinement. Refinement of *F*^2^ against ALL reflections. The weighted *R*-factor *wR* and goodness of fit *S* are based on *F*^2^, conventional *R*-factors *R* are based on *F*, with *F* set to zero for negative *F*^2^. The threshold expression of *F*^2^ > σ(*F*^2^) is used only for calculating *R*-factors(gt) *etc*. and is not relevant to the choice of reflections for refinement. *R*-factors based on *F*^2^ are statistically about twice as large as those based on *F*, and *R*- factors based on ALL data will be even larger.

### Fractional atomic coordinates and isotropic or equivalent isotropic displacement parameters (Å^2^)

**Table:**

	*x*	*y*	*z*	*U*_iso_*/*U*_eq_	
Cu1	0.85476 (4)	0.75682 (3)	1.00645 (2)	0.04079 (13)	
N1	0.6302 (3)	0.85640 (18)	0.93496 (14)	0.0382 (5)	
N2	0.7092 (3)	0.68048 (19)	1.05583 (16)	0.0432 (5)	
N3	1.1760 (3)	0.7822 (2)	1.16329 (17)	0.0455 (5)	
N4	0.9216 (4)	0.7086 (3)	0.8692 (2)	0.0665 (6)	
O1	1.0825 (3)	0.87642 (17)	1.14048 (15)	0.0557 (5)	
O2	1.0982 (3)	0.68978 (17)	1.10938 (14)	0.0517 (5)	
O3	1.3353 (3)	0.7747 (2)	1.23263 (17)	0.0651 (6)	
O4	0.8432 (3)	0.6241 (2)	0.88330 (17)	0.0742 (6)	
O5	0.9581 (3)	0.80226 (19)	0.92397 (15)	0.0522 (5)	
O6	0.9683 (4)	0.7076 (3)	0.8099 (2)	0.0924 (9)	
C1	0.6062 (4)	0.9480 (2)	0.87584 (19)	0.0459 (6)	
H1	0.7007	0.9662	0.8643	0.055*	
C2	0.4471 (4)	1.0162 (3)	0.83155 (19)	0.0496 (7)	
H2	0.4345	1.0798	0.7912	0.059*	
C3	0.3067 (4)	0.9886 (3)	0.8480 (2)	0.0500 (7)	
H3	0.1970	1.0328	0.8182	0.060*	
C4	0.3301 (4)	0.8943 (2)	0.90937 (19)	0.0438 (6)	
H4	0.2369	0.8749	0.9216	0.053*	
C5	0.4940 (3)	0.8294 (2)	0.95232 (17)	0.0365 (5)	
C6	0.5380 (4)	0.7277 (2)	1.02006 (18)	0.0377 (5)	
C7	0.4185 (4)	0.6821 (3)	1.0464 (2)	0.0491 (7)	
H7	0.3015	0.7159	1.0222	0.059*	
C8	0.4744 (5)	0.5857 (3)	1.1093 (2)	0.0584 (8)	
H8	0.3950	0.5538	1.1275	0.070*	
C9	0.6479 (5)	0.5371 (3)	1.1448 (2)	0.0634 (8)	
H9	0.6870	0.4716	1.1867	0.076*	
C10	0.7635 (4)	0.5873 (3)	1.1170 (2)	0.0557 (7)	
H10	0.8818	0.5555	1.1414	0.067*	

### Atomic displacement parameters (Å^2^)

**Table:**

	*U*^11^	*U*^22^	*U*^33^	*U*^12^	*U*^13^	*U*^23^
Cu1	0.03299 (19)	0.0431 (2)	0.0462 (2)	−0.00251 (13)	0.02050 (16)	−0.00080 (13)
N1	0.0358 (11)	0.0423 (10)	0.0365 (11)	−0.0015 (9)	0.0188 (10)	0.0014 (9)
N2	0.0410 (13)	0.0437 (12)	0.0431 (12)	−0.0026 (9)	0.0206 (11)	0.0018 (10)
N3	0.0402 (13)	0.0523 (13)	0.0418 (13)	−0.0059 (10)	0.0199 (11)	−0.0011 (10)
N4	0.0721 (14)	0.0727 (13)	0.0619 (12)	−0.0265 (11)	0.0400 (11)	−0.0237 (11)
O1	0.0553 (13)	0.0447 (11)	0.0573 (12)	0.0011 (9)	0.0224 (11)	−0.0035 (9)
O2	0.0386 (11)	0.0447 (10)	0.0592 (12)	−0.0014 (8)	0.0167 (10)	−0.0086 (9)
O3	0.0428 (13)	0.0776 (15)	0.0510 (13)	−0.0054 (10)	0.0077 (11)	0.0021 (11)
O4	0.0794 (14)	0.0738 (12)	0.0672 (12)	−0.0313 (10)	0.0366 (11)	−0.0219 (10)
O5	0.0488 (12)	0.0558 (11)	0.0627 (13)	−0.0111 (9)	0.0364 (11)	−0.0134 (10)
O6	0.107 (2)	0.119 (2)	0.0800 (19)	−0.0350 (19)	0.0689 (19)	−0.0397 (17)
C1	0.0490 (16)	0.0491 (15)	0.0432 (15)	−0.0057 (12)	0.0266 (14)	0.0033 (12)
C2	0.0590 (18)	0.0460 (15)	0.0401 (15)	0.0019 (13)	0.0234 (14)	0.0079 (12)
C3	0.0449 (16)	0.0477 (15)	0.0456 (15)	0.0049 (12)	0.0152 (14)	−0.0002 (13)
C4	0.0369 (14)	0.0487 (14)	0.0440 (14)	−0.0026 (11)	0.0198 (12)	−0.0014 (12)
C5	0.0343 (13)	0.0420 (13)	0.0314 (12)	−0.0063 (10)	0.0158 (11)	−0.0053 (10)
C6	0.0388 (14)	0.0393 (12)	0.0342 (13)	−0.0046 (10)	0.0185 (11)	−0.0013 (10)
C7	0.0463 (16)	0.0550 (16)	0.0514 (16)	−0.0056 (13)	0.0293 (14)	0.0022 (13)
C8	0.070 (2)	0.0595 (17)	0.0580 (19)	−0.0072 (16)	0.0421 (18)	0.0075 (15)
C9	0.081 (2)	0.0558 (17)	0.0531 (18)	−0.0018 (16)	0.0347 (18)	0.0170 (15)
C10	0.0571 (19)	0.0517 (16)	0.0519 (17)	0.0059 (14)	0.0239 (15)	0.0120 (14)

### Geometric parameters (Å, º)

**Table:**

Cu1—N1	1.966 (2)	C1—H1	0.9300
Cu1—N2	1.970 (2)	C2—C3	1.376 (4)
Cu1—O1	2.411 (2)	C2—H2	0.9300
Cu1—O2	1.994 (2)	C3—C4	1.385 (4)
Cu1—O4	2.437 (2)	C3—H3	0.9300
Cu1—O5	1.9987 (19)	C4—C5	1.383 (3)
N1—C1	1.338 (3)	C4—H4	0.9300
N1—C5	1.350 (3)	C5—C6	1.480 (3)
N2—C10	1.337 (3)	C6—C7	1.378 (3)
N2—C6	1.351 (3)	C7—C8	1.380 (4)
N3—O3	1.223 (3)	C7—H7	0.9300
N3—O1	1.247 (3)	C8—C9	1.374 (4)
N3—O2	1.285 (3)	C8—H8	0.9300
N4—O6	1.210 (3)	C9—C10	1.386 (4)
N4—O4	1.236 (3)	C9—H9	0.9300
N4—O5	1.296 (3)	C10—H10	0.9300
C1—C2	1.373 (4)		
			
N1—Cu1—N2	82.47 (9)	N1—C1—C2	122.5 (2)
N1—Cu1—O2	163.34 (8)	N1—C1—H1	118.8
N2—Cu1—O2	95.09 (9)	C2—C1—H1	118.8
N1—Cu1—O5	94.96 (9)	C1—C2—C3	118.7 (3)
N2—Cu1—O5	163.31 (9)	C1—C2—H2	120.6
O2—Cu1—O5	91.92 (9)	C3—C2—H2	120.6
N1—Cu1—O1	106.76 (8)	C2—C3—C4	119.4 (3)
N2—Cu1—O1	104.35 (8)	C2—C3—H3	120.3
O2—Cu1—O1	57.74 (8)	C4—C3—H3	120.3
O5—Cu1—O1	92.20 (8)	C5—C4—C3	119.2 (2)
N1—Cu1—O4	104.21 (9)	C5—C4—H4	120.4
N2—Cu1—O4	107.43 (8)	C3—C4—H4	120.4
O2—Cu1—O4	92.26 (9)	N1—C5—C4	121.0 (2)
O5—Cu1—O4	57.07 (8)	N1—C5—C6	114.3 (2)
O1—Cu1—O4	137.86 (8)	C4—C5—C6	124.8 (2)
C1—N1—C5	119.2 (2)	N2—C6—C7	121.0 (2)
C1—N1—Cu1	126.18 (17)	N2—C6—C5	114.4 (2)
C5—N1—Cu1	114.53 (16)	C7—C6—C5	124.6 (2)
C10—N2—C6	119.7 (2)	C6—C7—C8	119.3 (3)
C10—N2—Cu1	126.0 (2)	C6—C7—H7	120.4
C6—N2—Cu1	114.28 (17)	C8—C7—H7	120.4
O3—N3—O1	123.4 (3)	C9—C8—C7	119.6 (3)
O3—N3—O2	119.6 (2)	C9—C8—H8	120.2
O1—N3—O2	116.9 (2)	C7—C8—H8	120.2
O6—N4—O4	124.3 (3)	C8—C9—C10	118.8 (3)
O6—N4—O5	119.2 (3)	C8—C9—H9	120.6
O4—N4—O5	116.5 (2)	C10—C9—H9	120.6
N3—O1—Cu1	83.43 (15)	N2—C10—C9	121.6 (3)
N3—O2—Cu1	101.85 (16)	N2—C10—H10	119.2
N4—O4—Cu1	83.75 (17)	C9—C10—H10	119.2
N4—O5—Cu1	102.68 (17)		
			
N2—Cu1—N1—C1	178.0 (2)	N2—Cu1—O4—N4	172.5 (2)
O2—Cu1—N1—C1	95.5 (3)	O2—Cu1—O4—N4	−91.5 (2)
O5—Cu1—N1—C1	−18.6 (2)	O5—Cu1—O4—N4	−0.64 (19)
O1—Cu1—N1—C1	75.2 (2)	O1—Cu1—O4—N4	−50.3 (3)
O4—Cu1—N1—C1	−75.8 (2)	O6—N4—O5—Cu1	−179.7 (3)
N2—Cu1—N1—C5	0.66 (17)	O4—N4—O5—Cu1	−1.1 (3)
O2—Cu1—N1—C5	−81.9 (3)	N1—Cu1—O5—N4	−103.1 (2)
O5—Cu1—N1—C5	164.07 (17)	N2—Cu1—O5—N4	−22.8 (4)
O1—Cu1—N1—C5	−102.12 (17)	O2—Cu1—O5—N4	92.1 (2)
O4—Cu1—N1—C5	106.82 (17)	O1—Cu1—O5—N4	149.85 (19)
N1—Cu1—N2—C10	178.3 (2)	O4—Cu1—O5—N4	0.62 (19)
O2—Cu1—N2—C10	−18.3 (2)	C5—N1—C1—C2	−0.1 (4)
O5—Cu1—N2—C10	96.2 (4)	Cu1—N1—C1—C2	−177.4 (2)
O1—Cu1—N2—C10	−76.3 (2)	N1—C1—C2—C3	−0.5 (4)
O4—Cu1—N2—C10	75.7 (2)	C1—C2—C3—C4	0.8 (4)
N1—Cu1—N2—C6	0.38 (18)	C2—C3—C4—C5	−0.4 (4)
O2—Cu1—N2—C6	163.80 (18)	C1—N1—C5—C4	0.5 (3)
O5—Cu1—N2—C6	−81.7 (4)	Cu1—N1—C5—C4	178.04 (18)
O1—Cu1—N2—C6	105.83 (18)	C1—N1—C5—C6	−179.0 (2)
O4—Cu1—N2—C6	−102.22 (18)	Cu1—N1—C5—C6	−1.5 (3)
O3—N3—O1—Cu1	176.9 (2)	C3—C4—C5—N1	−0.2 (4)
O2—N3—O1—Cu1	−2.4 (2)	C3—C4—C5—C6	179.3 (2)
N1—Cu1—O1—N3	174.92 (14)	C10—N2—C6—C7	0.7 (4)
N2—Cu1—O1—N3	88.61 (15)	Cu1—N2—C6—C7	178.7 (2)
O2—Cu1—O1—N3	1.66 (14)	C10—N2—C6—C5	−179.3 (2)
O5—Cu1—O1—N3	−89.22 (15)	Cu1—N2—C6—C5	−1.3 (3)
O4—Cu1—O1—N3	−49.43 (19)	N1—C5—C6—N2	1.8 (3)
O3—N3—O2—Cu1	−176.4 (2)	C4—C5—C6—N2	−177.7 (2)
O1—N3—O2—Cu1	3.0 (2)	N1—C5—C6—C7	−178.2 (2)
N1—Cu1—O2—N3	−24.7 (4)	C4—C5—C6—C7	2.3 (4)
N2—Cu1—O2—N3	−105.41 (15)	N2—C6—C7—C8	−0.9 (4)
O5—Cu1—O2—N3	89.75 (15)	C5—C6—C7—C8	179.1 (3)
O1—Cu1—O2—N3	−1.63 (13)	C6—C7—C8—C9	0.3 (5)
O4—Cu1—O2—N3	146.86 (15)	C7—C8—C9—C10	0.6 (5)
O6—N4—O4—Cu1	179.4 (4)	C6—N2—C10—C9	0.2 (4)
O5—N4—O4—Cu1	0.9 (3)	Cu1—N2—C10—C9	−177.6 (2)
N1—Cu1—O4—N4	86.1 (2)	C8—C9—C10—N2	−0.8 (5)

### Hydrogen-bond geometry (Å, º)

**Table:**

*D*—H···*A*	*D*—H	H···*A*	*D*···*A*	*D*—H···*A*
C1—H1···O1^i^	0.93	2.56	3.390 (3)	149
C4—H4···O5^ii^	0.93	2.50	3.422 (3)	169

Symmetry codes: (i) −*x*+2, −*y*+2, −*z*+2; (ii) *x*−1, *y*, *z*.
